# Challenges Coexist with Opportunities: Spatial Heterogeneity Expression of PD‐L1 in Cancer Therapy

**DOI:** 10.1002/advs.202303175

**Published:** 2023-11-07

**Authors:** Yazhen Wang, Yang Zhou, Lianyi Yang, Lei Lei, Bin He, Jun Cao, Huile Gao

**Affiliations:** ^1^ National Engineering Research Center for Biomaterials College of Biomedical Engineering Sichuan University Chengdu 610064 P. R. China; ^2^ Key Laboratory of Drug‐Targeting and Drug Delivery System of the Education Ministry Sichuan Engineering Laboratory for Plant‐Sourced Drug and Sichuan Research Center for Drug Precision Industrial Technology West China School of Pharmacy Sichuan University Chengdu 610041 P. R. China

**Keywords:** cancer therapies, PD‐L1, regulatory mechanisms, small‐molecule modulators, spatial heterogeneity

## Abstract

Cancer immunotherapy using anti‐programmed death‐ligand 1 (PD‐L1) antibodies has been used in various clinical applications and achieved certain results. However, such limitations as autoimmunity, tumor hyperprogression, and overall low patient response rate impede its further clinical application. Mounting evidence has revealed that PD‐L1 is not only present in tumor cell membrane but also in cytoplasm, exosome, or even nucleus. Among these, the dynamic and spatial heterogeneous expression of PD‐L1 in tumors is mainly responsible for the unsatisfactory efficacy of PD‐L1 antibodies. Hence, numerous studies focus on inhibiting or degrading PD‐L1 to improve immune response, while a comprehensive understanding of the molecular mechanisms underlying spatial heterogeneity of PD‐L1 can fundamentally transform the current status of PD‐L1 antibodies in clinical development. Herein, the concept of spatial heterogeneous expression of PD‐L1 is creatively introduced, encompassing the structure and biological functions of various kinds of PD‐L1 (including mPD‐L1, cPD‐L1, nPD‐L1, and exoPD‐L1). Then an in‐depth analysis of the regulatory mechanisms and potential therapeutic targets of PD‐L1 is provided, seeking to offer a solid basis for future investigation. Moreover, the current status of agents is summarized, especially small molecular modulators development directed at these new targets, offering a novel perspective on potential PD‐L1 therapeutics strategies.

## Graphical abstract

In terms of PD‐L1's diverse expression, the concept of “spatial heterogeneity expression” is proposed to enhance comprehension of its tumor immune/non‐immune regulatory mechanism. Additionally, the structure, function, and regulatory mechanisms of PD‐L1 in various subcellular locations are summarized. Furthermore, potential modulators, including degraders, down regulators, and covalent inhibitors, are identified, providing valuable insights for future endeavors in drug development, delivery, and clinical therapy.

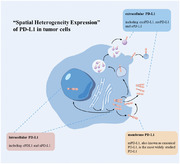

## 1 Introduction

Cancer immunotherapy possesses the potential to treat malignancy by mobilizing immune system. The invention of immunotherapy was revolutionary, as recognized with a Nobel Prize in 2018. The Nobel Prize committee awarded Tasuku Honjo for his main discovery of programmed death 1/programmed cell death ligand‐1 (PD‐1/PD‐L1), which caused a sensation from the oncology community in the coming years.^[^ ^1^ ^]^ PD‐L1 is a cell ligand that specifically binds to PD‐1 on T cells, leading to the inactivation of tumor killer T cells and the subsequent suppression of its anti‐tumor immune responses. Hence, the activation of the PD‐1/PD‐L1 pathway is associated with the tumor cell immune evasion mechanism. But PD‐L1 possesses far more research prospects than its receptor PD‐1 due to its spatial heterogeneity expression. Recent studies revealed that in addition to its localization in membrane, PD‐L1 also resides in cytoplasm and other subcellular structures.^[^ ^2^ ^]^ Hence, it exerts an impact on immune suppression function through a PD‐1‐dependent pathway, while also facilitating tumor invasion and proliferation via mechanisms independent of PD‐1.^[^ ^3^ ^]^ Thus, developing PD‐L1 small molecular modulators, such as degraders, down‐regulators, and covalent inhibitors, is essential to block related PD‐L1 processes and to reverse immunosuppression in tumor microenvironment.^[^ ^4^ ^]^

**Figure 1:**
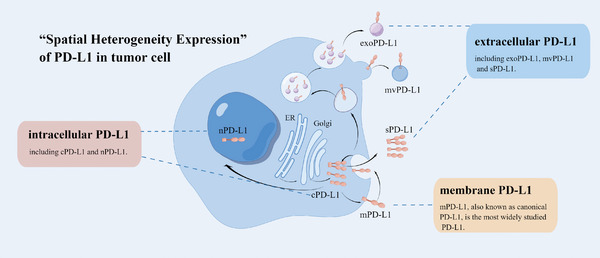
Schematic illustration of “spatial heterogeneity expression” of PD‐L1. PD‐L1 is synthesized in the endoplasmic reticulum (ER) and then modified in the Golgi apparatus. Then they either reside in the cytoplasm (cPD‐L1), move into the nucleus (nPD‐L1), secrete to the extracellular space (sPD‐L1), or transport to the cell membrane (mPD‐L1). cPD‐L1 and nPD‐L1 are described collectively as intracellular PD‐L1. mPD‐L1 also could endocytose into the cytoplasm and secrete exosomes into the extracellular milieu (exoPD‐L1). Beyond that, mPD‐L1 forms microvesicles by direct outward budding (mvPD‐L1). PD‐L1 can be in a highly dynamic state.

Anti‐PD‐L1 antibodies have been in the pharmaceutical pipeline since 2016.^[^ ^5^ ^]^ Since then, more antibodies targeting PD‐L1 have become commercially available, including atezolizumab, avelumab, durvalumab, and envolizumab, which have gained certain therapeutic effects.^[^ ^6^ ^]^ The efficacy of anti‐PD‐L1 therapy has been reported to be positively correlated with the expression of membrane PD‐L1 (mPD‐L1) on tumor cells.^[^ ^7^ ^]^ Based on this conclusion, the principal mechanism of PD‐L1 antibodies mentioned above is to bind with mPD‐L1, thus resuming the normal tumor‐killing functions of T cells.^[^ ^8^ ^]^ However, the response rates toward a monotherapy of PD‐L1 inhibition rarely exceed 30%,^[^ ^9^ ^]^ suggesting that solely inhibiting mPD‐L1 is inadequate to achieve satisfactory clinical efficacy. In addition, recent studies indicated that the spatial heterogeneity expression of PD‐L1 in tumor cells seemed more responsible for the unsatisfactory efficacy of anti‐PD‐L1 antibodies.^[^ ^10^ ^]^ In recent years, emerging studies have demonstrated that PD‐L1 is not only expressed on the tumor cell membranes but also broader cellular substructure of tumor cells, such as cytoplasm, nucleus and extracellular vesicles, etc.^[^ ^11^ ^]^ Moreover, PD‐L1 can also be recycled between the cytoplasm and the membrane (**Figure** **1**). mPD‐L1 enters the cytosol via huntingtin interacting protein 1‐related (HIP1R)^[^ ^12^ ^]^ while cytoplasmic PD‐L1 (cPD‐L1) is recycled to the cell membrane by trafficking protein particle subunit 4 (TRAPPC4).^[^ ^13^ ^]^ Simultaneously, exosomal PD‐L1 (exoPD‐L1) can be secreted from the primary tumor to other tissues to suppress tumor‐killing effects comprehensively.^[^ ^14^ ^]^ Owning to the heterogeneity of its expression levels, it is necessary to understand the subcellular distribution and inner mechanisms that regulate PD‐L1 expression in tumor cells, thus aiding the development of more targeted clinical treatment strategies.

In this review, we aim to provide a comprehensive understanding of the spatial heterogeneity expression of PD‐L1, encompassing its subcellular distribution, structure, function, and regulatory mechanisms. By exploring the multifaceted roles of PD‐L1 in cancer progression, including its participation in immunological/non‐immunological functions, there is a strong desire to enhance the advancement of more efficacious therapeutic approaches against tumors.^[^ ^15^ ^]^ Furthermore, by capitalizing on the aforementioned mechanisms, we underscore the potential for modulating PD‐L1 expression and/or PD‐L1/PD‐1 signaling through the use of small molecule modulators. The rational utilization of small molecule modulators and conventional antibody therapeutics holds greater promise for cancer treatment. Overall, this review article aims to shed light on the latest information on the PD‐L1 mechanisms in various subcellular compartments and their alternative therapeutic strategies targeting PD‐L1, especially small molecular modulators, providing a scholarly reference for researchers and clinicians seeking a deeper understanding of PD‐L1 signaling regulation. Ultimately, our objective is to stimulate further exploration in this booming field and advance our knowledge and management of cancer.

## 2 PD‐L1 Structure and its Function

**Figure 2:**
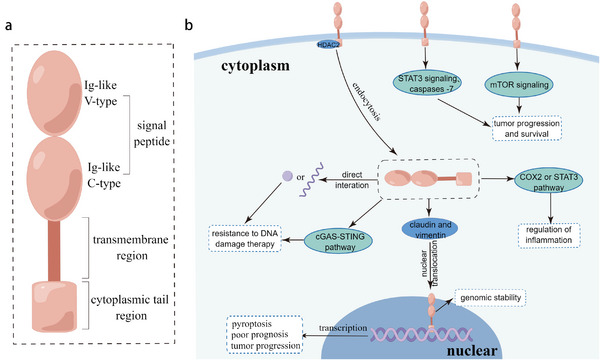
The intracellular function of PD‐L1 in cancer cells. a) The structure of full‐length PD‐L1. It contains a signal peptide, transmembrane region, and cytoplasmic tail region. b) The mPD‐L1 and cPD‐L1 intracellular signaling pathways and function. Considering that the whole situation is complex, we have not differentiated the structures of PD‐L1 at different locations, and instead, employed the same full‐length PD‐L1 structure to represent all PD‐L1.

Various subcellular locations of PD‐L1 exhibit different conformations. Generally, full‐length PD‐L1 is a 290 amino acid, type I transmembrane glycoprotein belonging to the immunoglobulin (Ig) superfamily. It contains one extracellular region that is composed of a signal peptide, an Ig‐like V‐type domain and two Ig‐like C‐type domains, one transmembrane region and one cytoplasmic tail region (**Figure** **2**a).^[^ ^1b^ ^]^ Among them, mPD‐L1 usually has full‐length amino acid sequences and complex tertiary conformations.^[^ ^16^ ^]^ But the domain of cPD‐L1 is usually partially lacking, for example, transmembrane domain deletion and N‐terminal domain deletion. Due to its spatial heterogeneity expression, PD‐L1 also plays a specific role in particular subcellular structures. Canonical PD‐L1, which refers to mPD‐L1, often exists as a transmembrane protein and functions as a PD‐1 ligand, which binds to PD‐1 on the cytotoxic T lymphocytes membrane to inhibit tumor immune responses. In other words, mPD‐L1 mainly regulates cancer immune activity in a PD‐1‐dependent manner. The non‐canonical forms of PD‐L1, specifically cPD‐L1, nPD‐L1, and exoPD‐L1, exhibit distinct biological functions that are correlated closely with tumor cell proliferation, immune response, DNA damage response, and gene expression control.^[^ ^17^ ^]^ And these effects include both PD‐1 dependent pathway and PD‐L1 independent pathway. The overall situation is complex, therefore, we did not specifically highlight which structure corresponds to which function. Hence, apart from the schematic in Figure 1, we employed the same full‐length PD‐L1 structure to represent PD‐L1 in different locations in other figures. In this section, we provide an overview of the recent progress in understanding the structure and functions of PD‐L1.

### 2.1 The Structure and Function of mPD‐L1

Considering that PD‐L1/PD‐1 interaction needs complex 3D structure, mPD‐L1 requires all regions mentioned above to form mature functional membrane protein.^[^ ^16^ ^]^ Among them, the Ig‐like V‐type extracellular region of mPD‐L1 can bind to PD‐1 or other receptors, thus leading to the inhibition or activation of its downstream signaling. Besides, the Ig‐like V‐type domain is also the binding site of Anti‐PD‐L1 monoclonal antibodies, so the Ig‐like V domain is an important therapeutic target.^[^ ^16^ ^]^ And the cytoplasmic tail region of mPD‐L1 contains a protein kinase C (PKC) phosphorylation site, but this whole signal transduction pathway to the intracytoplasmic tail is currently unknown.

**Figure 3:**
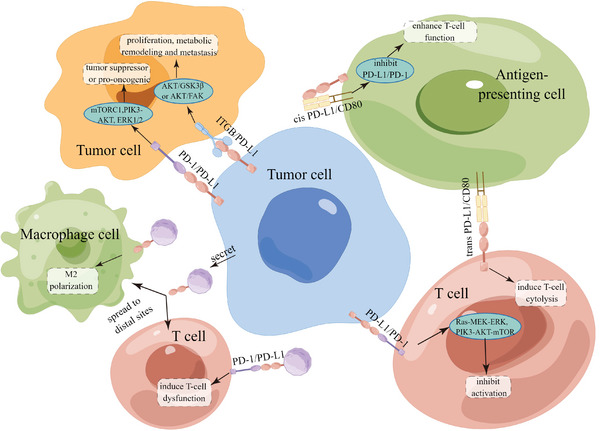
mPD‐L1 recognition by PD‐1 or other receptors triggers cellular signaling pathways that activate common signaling pathways in neighboring cells.

In terms of functions, mPD‐L1 expressed in tumor cells can specifically bind to PD‐1, expressed either in the T cell membrane or tumor cells, to induce downstream PD‐1 signaling pathway and cause tumor immune escape (**Figure** **3**). Therein, PD‐1 is a member of the CD28 superfamily that is predominantly expressed in activated T cells, including T regulatory cells, B cells, and myeloid cells.^[^ ^18^ ^]^ The classical function of PD‐L1, which is mainly mediated by mPD‐L1, involves the specific interaction between PD‐L1 and PD‐1 on T cells. This interaction promotes immune evasion in tumors. After binding, PD‐1 shifts to an active conformation, leading to the phosphorylation of its immunoreceptor tyrosine‐based inhibitory and switch motifs (lTIM and ITSM, respectively). The ITIM/ITSM domains then recruit the Src homology 2 domain containing phosphatases 1/2 (SHP1/2), which act as protein tyrosine phosphatases and are capable of inhibiting T‐cell activation signaling pathway,^[^ ^19^ ^]^ including the Ras‐MEK‐ERK pathway and the PlK3‐AKT‐mTOR pathway.^[^ ^20^ ^]^ Except for binding to PD‐1 on T cells, mPD‐L1 can also bind to PD‐1 on tumor cells.^[^ ^21^ ^]^ The binding between PD‐1 on melanoma cells and mPD‐L1 can activate the mTORC1 signaling pathway, resulting in increased tumor aggressiveness.^[^ ^21a^ ^]^ However, in NSCLC and colon cancer cells, the binding to them can inhibit tumor growth by activating PI3K‐AKT and ERK1/2 pathways.^[^ ^21b^ ^]^ Since these studies reported different functions of mPD‐L1 in different cell lines, it highlights the need for further research to clarify these inconsistencies. The reasons for such inconsistency may be complex and multifaceted, involving specific genetic backgrounds, biological processes, and post‐translational modifications.

Moreover, mPD‐L1 can also bind to non‐PD‐1 proteins, such as antigen differentiation cluster 80 (CD80)^[^ ^22^ ^]^ and integrin,^[^ ^23^ ^]^ and result in varying impacts on tumor growth (Figure 3). CD80 is usually present at the cellular surface of antigen‐presenting cells (APC) and is the major ligand mediating cytotoxic T‐lymphocyte‐associated antigen 4 (CTLA‐4), another important immune checkpoint protein.^[^ ^24^ ^]^ A previous study has shown that mPD‐L1 can interact with CD80 either within the same cell (cis‐interaction) or between adjacent cells (trans‐interaction), yielding completely different biological behaviors.^[^ ^25^ ^]^ The cis‐mPD‐L1/CD80 interaction inhibits them from binding to their respective targeting proteins, PD‐1 and CTLA‐4, subsequently eliminates immune brakes and activates T cells.^[^ ^22a^ ^]^ But trans‐mPD‐L1/CD80 interaction is found to cause the inhibition of T‐cell activation, leading to reduced cell proliferation and cytolysis.^[^ ^26^ ^]^ Otherwise, integrin, mainly integrin β4 (ITGB4) and integrin β6 (ITGB6), can also bind to mPD‐L1 and then activate the Protein kinase B (AKT)/ glycogen synthase kinase 3β (GSK3β) pathway and AKT/FAK signaling pathways, resulting in cell migration and invasion capabilities.^[^ ^23^ ^]^ These findings are direct evidence for the PD‐1‐independent manner of mPD‐L1. Overall, this PD‐1 independent manner turns our attention from the single “tumor cell to T cells” manner to “tumor cell to tumor cell” or another different cell manner.

Most literature surveyed above focused on mPD‐L1 binding to PD‐1 or other receptors on neighboring cells, leading to the activation of common signaling pathways.^[^ ^22^, ^23^ ^]^ However, it should be recognized that mPD‐L1 is also capable of triggering tumor‐intrinsic signaling pathways (Figure 2b). Recent studies have shown that the cytoplasmic C‐tail of mPD‐L1 could enhance tumor cell survival by preventing apoptosis and increasing the stability of mPD‐L1 itself. First, mPD‐L1 promotes tumor progression and inhibits apoptosis by preventing cytotoxic effects of IFN on tumor cells through the inhibition of the STAT3/caspase‐7 signaling pathway.^[^ ^27^ ^]^ Additionally, mPD‐L1 can also inhibit tumor cell apoptosis by suppressing tumor autophagy and activating the mTOR pathway.^[^ ^28^ ^]^ Second, studies have also demonstrated that the cytoplasmic domain of mPD‐L1 is critical in regulating the stability of mPD‐L1 and mediating the immune escape of tumor cells. The cytoplasmic domain of the mPD‐L1 can be acetylated by acetyltransferase p300 at Lys263, while deacetylated by histone deacetylase 2 (HDAC2).^[^ ^17b^ ^]^ The deacetylated cytoplasmic domain of mPD‐L1 enhances the interaction with endocytic trafficking proteins, such as HIP1R, and triggers nuclear translocation.^[^ ^12^ ^]^ Furthermore, the basic residues in the cytoplasmic tail of mPD‐L1 interact with acidic phospholipids on the cell membrane, thus regulating the conformation of mPD‐L1 and enhancing its stability.^[^ ^29^ ^]^ Overall, the intracellular regions of mPD‐L1 mainly regulate the growth of tumors by activating intracellular signaling pathway alone.

### 2.2 The Structure and Function of cPD‐L1

Since its detection in collected circulating tumor cells (CTCs) by immunofluorescence, a growing body of research have been performed, illustrating cPD‐L1 location and roles.^[^ ^17^, ^30^ ^]^ Unlike mPD‐L1 which primarily localizes on the cell membrane, cPD‐L1 is found in the inner part of tumor cells, potentially being protected from binding by current PD‐L1 antibodies. Thus, further investigation is required to better understand the structure and function of cPD‐L1.

As previously reviewed, full‐length PD‐L1 was composed of an extracellular region, a transmembrane region, and a cytoplasmic tail region. When PD‐L1 is situated in the cytoplasm, the extracellular region of the protein remains largely unfolded, while the Ig‐V and Ig‐C domains of cPD‐L1 participate in other signal transduction mechanisms that are independent of PD‐1. Despite its potential importance, the isoform of cPD‐L1 has received little attention in the literature. This neglect of the cPD‐L1 isoform in the literature underscores the need for future studies that specifically investigate its structure.

By far, the main functions of cPD‐L1 are unrelated to the immune system. This non‐immune function of cPD‐L1 is crucial in regulating several cellular processes that promote tumor growth and resistance to anti‐cancer therapies. cPD‐L1 regulates tumor cell proliferation, apoptosis, and resistance to chemotherapy and radiotherapy.^[^ ^3^, ^31^ ^]^ One of the critical functions of cPD‐L1 is its ability to protect tumor cells from the cytotoxic effects of chemotherapy and radiotherapy. Mechanistically, cPD‐L1 can bind to DNA damage‐related mRNAs, such as NBS1 and BRCA1 to prevent these RNAs from degradation, thereby reducing DNA damage‐induced cell death.^[^ ^31^ ^]^ Additionally, cPD‐L1 also sustains constitutive activation of the cGAS‐STING pathway and the expression of low levels of IFN‐β, which induces pro‐survival IRDS expression (IRDS is a highly upregulated gene in various cancer types that is often associated with the resistance to chemotherapy or radiation),^[^ ^3a^ ^]^ and subsequently leading to drug resistance. Besides playing its role in protecting tumor cells from chemo‐ and radiotherapy, cPD‐L1 also participates in the process of tumor resistance triggered by Breast Cancer Susceptibility Protein‐1 (BRCA1), which regulates DNA repair through homologous recombination. cPD‐L1 promotes BRCA1 nuclear foci formation and DNA end resection, which is involved in resistance to chemotherapy and radiotherapy.^[^ ^3b^ ^]^ These mechanisms contribute to the development of drug resistance to some extent.

cPD‐L1 not only helps tumor cells evade the cytotoxic effects of chemotherapy and radiotherapy, but also modulates several inflammatory responses, including COX2 and STAT3, which promote the survival and growth of tumors. Tumor development is closely associated with inflammatory reactions that involve in various signaling pathways.^[^ ^32^ ^]^ An earlier study has indicated that anti‐inflammatory medicine, such as resveratrol, could inhibit constitutive cPD‐L1 expression and accumulation in tumor cells, thus inhibiting cell proliferation, suggesting that cPD‐L1 is involved in inflammatory effect on cancer.^[^ ^33^ ^]^ Initial mechanism studies showed that cPD‐L1 is regulated by the STAT3 signaling pathway and nuclear translocation of Cyclooxygenase 2 (COX‐2), which promotes tumor cell proliferation.^[^ ^34^ ^]^ However, the molecular mechanisms by which cPD‐L1 exerts its proliferation function need to be further elucidated.

Taken together, the non‐immune function of cPD‐L1 also plays a critical role in DNA damage repair and tumor progression. However, limited attention is focused on these non‐immune functions. Further studies are necessary to identify the underlying mechanisms of cPD‐L1 regulation and its involvement in tumor development and resistance to anti‐cancer therapies.

### 2.3 The Structure and Function of nPD‐L1

Through nuclear translocation, cPD‐L1 is shuttled from the cytoplasm into the nucleus, resulting in the formation of nPD‐L1, which has been observed in CTCs collected from patients with prostate, breast, and colorectal cancer, as well as osteosarcoma.^[^ ^35^ ^]^ Since the related structure of nPD‐L1 is not reported now, our subsequent discussion will only focus on its functional properties, including its role in regulating gene expression, promoting proliferation and predicting prognosis.

First, nPD‐L1 can enhance the expression of immune‐related and pro‐inflammatory‐related genes in tumor cells, leading to tumor progression. In CD247‐(encoding PD‐L1 gene) knockdown MDA‐MB‐231 cells, RNA sequencing and chromatin immunoprecipitation sequencing revealed that nPD‐L1 present coincidence peak with 60% downregulated genes, including programmed death ligand 2 (PD‐L2), V‐domain immunoglobulin suppressor of T cell activation (VISTA), and B7 homolog 3 (B7‐H3). This suggested that the expression of PD‐L1‐dependent genes is mediated through nPD‐L1 interaction with DNA or with DNA‐bound transcription factors, functioning as a transcription factor to modulate immune function‐associated gene expression.^[^ ^17b^ ^]^

nPD‐L1 can also serve as a key nuclear protein that promotes immune‐independent cell proliferation without binding to PD‐1.^[^ ^36^ ^]^ High expression of nPD‐L1 in the nucleus can promote tumor cell proliferation and growth. Further mechanistic investigation has revealed that cPD‐L1 is translocated into the nucleus via p‐ERK activation, which then triggers the expression of BUB1, a type of cell‐cyclin controlling protein, by binding to thyroid hormone receptor‐associated protein 3 (THRAP3), thus facilitating cell cycle progression and promoting cell proliferation.

nPD‐L1 expression is closely associated with tumor progression and poor prognosis. Karyopherin‐β1 (KPNB1) has been shown to mediate cPD‐L1 translocation to the nucleus, thus activating the Gas/MerTk signaling pathway and promoting the proliferation of NCSLC.^[^ ^17a^ ^]^ nPD‐L1 can bind to transcription factor Sp1 and promote Gas6 transcription. Subsequently, the newly produced Gas6 is secreted into extracellular space and bound to membrane Tyrosine‐protein kinase Mer (MerTK), activating the oncogenic RAS/ERK and PI3K/AKT signaling pathway and thereby regulating cell proliferation and survival. Otherwise, high expression of nPD‐L1 is significantly correlated with shorter overall survival among patients with prostate and colorectal cancer.^[^ ^35^ ^]^ Overall, these non‐immune functions of nPD‐L1 also promote tumor progression.

### 2.4 The Structure and Function of Extracellular PD‐L1

As mentioned above, PD‐L1 has variable localization, which can be either on the cell surface, within the cytoplasm, within the nucleus, or secreted to extracellular space. Several different forms of extracellular PD‐L1 have been identified in the extracellular site such as supernatants from cell cultures and human serum, including exoPD‐L1, microvesicular PD‐L1 (mvPD‐L1), and soluble PD‐L1 (sPD‐L1, which contains monomeric shed form of PD‐L1 and dimeric splice variants of PD‐L1) (Figure 1).^[^ ^37^ ^]^ In breast cancer, most of the synthesized PD‐L1 protein is secreted into the distal tissues (in exoPD‐L1 form), which makes PD‐L1 rarely present in intracellular space.^[^ ^38^ ^]^ Meanwhile, sPD‐L1 can also be detected in renal cell carcinoma and NSCLC.^[^ ^37^, ^39^ ^]^ Emerging evidence suggests that signals transmitted by this exogenous PD‐L1 to other cells are likely to be relevant to tumor progression. In this part, we present the structure and function of extracellular PD‐L1 in detail.

In terms of structure, extracellular PD‐L1 has both full‐length form and short forms. Since exosome is a vesicle derived from the host cell membranes, it possesses phospholipid bilayer membranes. Similarly, exoPD‐L1 has the same membrane topology as mPD‐L1, with its extracellular domain exposed on the surface of the exosomes (details are given Section in 2.1).^[^ ^40^ ^]^ But the situation is different in sPD‐L1. Specifically, sPD‐L1 can be further divided into monomeric, dimeric splice variants, and cleaved monomeric shed forms of PD‐L1. They are usually formed from the splicing or dimerization, or both, of cPD‐L1. Various types of sPD‐L1 isoforms, which lack transmembrane domain, are identified in a melanoma model and in patients with NSCLC.^[^ ^39^, ^41^ ^]^ Besides, another sPD‐L1 is also characterized for lacking transmembrane domain and dimerization of cytoplasmic C‐tail domain.^[^ ^42^ ^]^ These findings have significantly improved our grasp of structural variation of extracellular PD‐L1.

As for its function, extracellular PD‐L1 is associated with antitumor immune response. In the following, we summarize the function of extracellular PD‐L1, including exoPD‐L1 and sPD‐L1. Current studies suggest that exoPD‐L1 not only plays a critical role in inducing immune evasion but also functions as a potential biomarker in predicting the clinical response to anti‐PD‐1/PD‐L1 immunotherapies.^[^ ^43^ ^]^ On the one hand, exoPD‐L1 is secreted to distal tissues by cancer cells,^[^ ^38^ ^]^ and then attaches to PD‐1 on the surface of T cells, which in turn suppresses and tyrannizes the immune system.^[^ ^40^, ^44^ ^]^ On the other hand, exoPD‐L1 is found to effectively impede the activation and proliferation of T cells, and then exhibit a certain against anti‐PD‐L1 antibody blockade to some extent,^[^ ^45^ ^]^ which can be reversed through the administration of anti‐PD‐1 treatment in glioblastoma and various other cancer types.^[^ ^46^ ^]^

In addition to suppressing T cell functions, exoPD‐L1 may act on the polarization of macrophages. It can polarize tumor‐associated macrophages (TAMs) into the tumor‐promoting M2 type and upregulate the expression of PD‐L1 in TAMs.^[^ ^47^ ^]^ The increasing exoPD‐L1 secretion parallels the increasing M2 macrophage polarization, thus rendering a tumor microenvironment favorable for its growth and survival. Golgi membrane protein 1 (GOLM1) has been found to be positively correlated with infiltrating TAMs expressing high levels of PD‐L1 and CD8 T cell suppression in HCC tissues.^[^ ^48^ ^]^ GOLM1 can regulate the exocytosis activities by promoting the cargo transportation of exosomes, which will promote the delivery of PD‐L1 to the macrophages equipped with the exosome carrier. In this mechanism, GOLM1 promotes COP9 signalosome 5‐mediated PD‐L1 deubiquitination in HCC cells and increases the transport of PD‐L1 into exosomes via suppression of Rab27b expression. Additionally, a previous study demonstrated that endoplasmic reticulum (ER) stress‐induced oral squamous cell carcinoma (OSCC) cells secreted exoPD‐L1 that resulted in upregulating PD‐L1 expression in macrophages and driving M2 macrophage polarization.^[^ ^49^ ^]^ Furthermore, the co‐cultivation of macrophages with exosomes derived from HCC cells can upregulate the expression of PD‐L1 on macrophages.

ExoPD‐L1 can also lead to resistance to chemotherapeutic drugs and function as an indicator of clinical prognosis. ExoPD‐L1 can enhance the resistance of esophageal cancer cells to paclitaxel by promoting the transcription of miRNA21 by inducing STAT3 into the nucleus.^[^ ^50^ ^]^ Notably, exoPD‐L1, rather than other extracellular forms, might serve as a biomarker for predicting tumor progression and immune checkpoint inhibitor response. A few publications have shown that exoPD‐L1 is a marker of poor outcomes after surgery or chemotherapy and radiation in patients with gastric cancer and head and neck squamous cell carcinoma (HNSCC).^[^ ^37b^ ^]^

Apart from exoPD‐L1, sPD‐L1 can also compromise T‐cell viability and confer immunotherapy resistance. A type of sPD‐L1, isolated from serum in clear cell renal carcinoma patients, potently suppressed T‐cell function by inducing T‐cell apoptosis or blocking CD4^+^ T cells and CD8^+^ T cells responses.^[^ ^37a^ ^]^ Another sPD‐L1, found in serum from patients with stage 5 malignant melanoma, predicted the prognosis of melanoma patients receiving immune‐checkpoint inhibitors, including PD‐1 and CTLA‐4 inhibitors.^[^ ^41^ ^]^ Moreover, sPD‐L1 tends to homodimerize, which performs more effectively in inhibiting T lymphocyte activation than monomeric sPD‐L1.^[^ ^51^ ^]^ Additionally, sPD‐L1 can trap all PD‐L1 antibodies in NSCLC patients^[^ ^39^ ^]^ and modulate regulatory B cells differentiation and then weaken the anti‐neoplastic immune response ability of T cells.^[^ ^52^ ^]^ These studies revealed that sPD‐L1 has an unique mechanism of action different from other forms of PD‐L1.

## 3 mPD‐L1 Expression Regulation Mechanisms and Therapeutic Strategies for Tumor Therapy

mPD‐L1 is the first discovered immune checkpoint that results in tumor cell proliferation and metastasis, so the blockage of the mPD‐L1 has shown excellent antitumor efficacy in a variety of solid tumors. In the following part of this review, we first describe the relevant information on the mPD‐L1 expression regulatory mechanism, then we identify potential drug targets and review the mPD‐L1 modulators that have been reported. Among them, small‐molecule agents account for the majority and are expected to be in a new position for the development of tumor drugs.

### 3.1 Regulation of mPD‐L1 Expression

#### 3.1.1 Mechanisms for Up‐Regulation of mPD‐L1

mPD‐L1, like other proteins, does not always exist at a high level and undergoes the synthesis of new ones and the degradation of old ones. Considering the biosynthesis of mPD‐L1 occurs at an earlier stage than degradation, we first elaborate on the up‐regulation mechanisms. On the one hand, newly synthesized PD‐L1 is transported from the Golgi apparatus to membrane.^[^ ^53^ ^]^ On the other hand, mPD‐L1 conducts a series of measures to save itself from degradation. Post‐transnational modifications involving ubiquitination, acetylation, and palmitoylation reactions can control mPD‐L1's stability and transcriptional activity. Besides, several related proteins, cholesterol, or acidic phospholipids in cell membranes can also regulate the stability of mPD‐L1.

First, the synthesis and maturation of mPD‐L1 is a multistep process which is finely regulated. During the whole process, synthesized PD‐L1 in the cytosol is transported to the membrane to restore the mPD‐L1 pool, thus promoting immune evasion.^[^ ^53^, ^54^ ^]^ Specifically, newly synthesized PD‐L1 is added with k63‐linked ubiquitination under the control of mind bomb homolog 2 (MIB2), a type of E3 ligase. Then ubiquitinated PD‐L1 translocates from Golgi to the membrane by Ras‐associated binding 8‐mediated (RAB8‐mediated) exocytosis.^[^ ^53^ ^]^ However, little is known about the mechanism driving PD‐L1 translocation to the plasma membrane after de novo synthesis. More efforts should be directed toward the mechanism of de novo synthesis.

Then, to avoid elimination by immune cells, tumor cells apply a series of strategies to stabilize mPD‐L1. In some conditions, tumor cells can reduce the ubiquitination labeling of mPD‐L1 to minimize its degradation, leading to immune escape. Given that ubiquitination labeling is a major mechanism to down‐regulate protein, this aspect will be elaborated on later in “Mechanism for Down‐Regulation of mPD‐L1”. In addition to its effects on the degradation of mPD‐L1, tumor cells also affect the biosynthesis process. A series of extensive post‐translational modifications, including acetylation and palmitoylation, or interaction with other proteins contribute partly to mPD‐L1's stability, as described below.

Acetylation modification of mPD‐L1 can affect the stability or subcellular localization of mPD‐L1.^[^ ^17b^ ^]^ It is the process of adding acetyl groups (CH_3_CO‐) to lysine residues on the protein chain, which is a reversible process. A previous study identified that the histone acetyltransferase p300 acetylates mPD‐L1 at Lys 263 while histone deacetylase 2 (HDAC2) deacetylates mPD‐L1. The acetylated mPD‐L1 can prevent itself from spreading to the cytoplasm and nucleus. In other words, acetylation modification stabilizes mPD‐L1. In comparison to this, deacetylated mPD‐L1 tends to bind to HIRP1 and promotes clathrin‐mediated endocytosis, thus triggering nuclear translocation under the influence of vimentin and importin‐α.^[^ ^17b^ ^]^ In addition, mammalian hepatitis B X‐interacting protein (HBXIP) can also initiate acetylation modification of mPD‐L1 through p300 in breast cancer cells.^[^ ^55^ ^]^ Specifically, HBXIP interacts with p300 and then induces the PD‐L1 acetylation at the K270 site.

Palmitoylation modification can enhance the mPD‐L1 anchoring of the target proteins and influence its stability. It is a post‐translational modification process that conjugates palmitic acid, a 16‐carbon lipid, to specific cysteine residues. Three independent studies have shown that mPD‐L1 is palmitoylated at C272 in its cytoplasmic domain by palmitoyltransferase ZDHHC3 (DHHC3). And this modification stabilizes mPD‐L1 by preventing its ubiquitination, which in turn inhibits its subsequent lysosomal degradation.^[^ ^56^ ^]^ Moreover, mPD‐L1 binds acidic phospholipids via its N‐terminal domain and increases its stability. It has a preferred location in an acidic lipid‐rich environment.^[^ ^29^ ^]^ The exact mechanism is that three arginines in the membrane‐proximal region, R260, R262, and R265, bind to the acidic phospholipids through electrostatic interaction, therefore inhibiting post‐translational modification and downstream degradation.^[^ ^29^ ^]^

In addition to palmitoylation and acetylation modifications, mPD‐L1 can bind to other membrane proteins to protect itself against degradation. CKLF‐like MARVEL transmembrane domain‐containing protein 6 (CMTM6), a protein widely expressed in cell‐surface, is a master “positive” regulator for mPD‐L1. In the plasma membrane and recycling endosomes, CMTM6 binds directly to PD‐L1 to stabilize mPD‐L1 by preventing its STUB1‐mediated polyubiquitination and degradation.^[^ ^57^ ^]^ CMTM4, another CMTM family member that is closely related to CMTM6, is also identified as a positive regulator of mPD‐L1 in the absence of CMTM6.^[^ ^57a^ ^]^ It is worth noting that CMTM6 and CMTM4 only act as stabilizers of mPD‐L1 at the cell surface, namely at the protein level following biosynthesis, and are not involved in PD‐L1 maturation. Besides, cholesterol, as an essential component of a biological membrane, was also proven to stabilize mPD‐L1.^[^ ^58^ ^]^ Two CRAC motifs in the mPD‐L1 transmembrane region bind with cholesterol to form a sandwich‐like architecture, thus enhancing mPD‐L1 stability. However, it is currently unclear which signaling pathways are responsible for modulating mPD‐L1 levels through cholesterol. Further research is needed to fully elucidate the mechanisms involved.

#### 3.1.2 Mechanisms for Down‐Regulation of mPD‐L1

**Figure 4:**
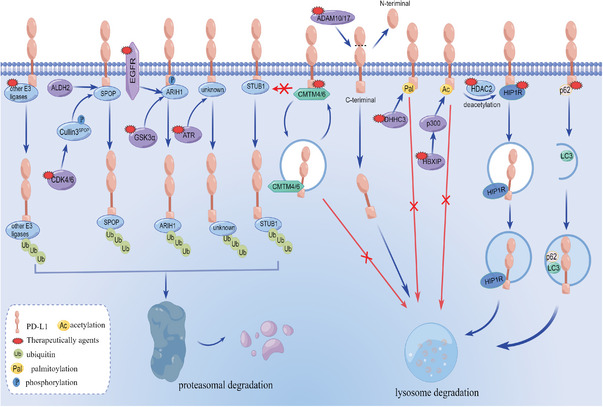
mPD‐L1 regulation mechanisms and therapeutic strategies. mPD‐L1 expression level is continuously fluctuating, with mechanisms in place to stimulate or suppress synthesis based on physiological demands. Within the context of normal physiology, mPD‐L1 can be down‐regulated by degradation pathways facilitated by ubiquitin enzymes (blue ovals) and related proteins (dark blue ovals). But tumor cells can invoke various mechanisms to up‐regulate mPD‐L1, such as promoting its synthesis and inhibiting its degradation (purple ovals, yellow little ovals, and green polygons). Besides, the red arrows represent the prevention of degradation processes and the small red polygons denote small molecular modulators targeting the corresponding targets.

In contrast to the up‐regulation mechanisms, more research about the down‐regulation mechanisms of mPD‐L1 is revealed. This is mainly because the down‐regulated mPD‐L1 can attenuate tumor immune evasion and dampen cancer progression. During the remodeling process of mPD‐L1, ubiquitination is a crucial process that should not be ignored. Ubiquitination is an enzymatic process that involves the attachment of ubiquitin and the degradation of labeling cellular proteins by the proteasome and the lysosomal machinery (**Figure** **4**).^[^ ^59^ ^]^ In the whole process, ubiquitin is activated by the E1 ubiquitin‐activating enzyme and transferred to E2 ubiquitin‐conjugating enzymes. Then E3 ubiquitin ligase brings the substrate, mPD‐L1, into proximity of an E2 ubiquitin‐conjugating enzyme to promote substrate ubiquitylation. After covalently labeled with ubiquitin chains, mPD‐L1 was recognized and degraded by the proteasome or lysosome system. Notably, E3 ligase determines the specificity of substrate‐here refers to mPD‐L1.^[^ ^60^ ^]^ Currently, several E3 ligases, such as cullin3^SPOP^, STUB1, ARIH1, and ITCH, have been identified to mediate the degradation of mPD‐L1 according to various mechanisms.^[^ ^61^ ^]^

SPOP is the first identified and the most widely‐studied E3 ubiquitin ligase to mediate PD‐L1 degrading. The detailed mechanism of PD‐L1 ubiquitination and degradation is based on the cyclinD‐dependent kinases 4 (CDK4) /SPOP/Cdh1 pathway.^[^ ^61c^ ^]^ Specifically, CDK4 induces SPOP phosphorylation and promotes the Cullin3^SPOP^ dissociation. The dissociated SPOP thus activates polyubiquitination degradation of PD‐L1, thus inducing downregulation of mPD‐L1. Additionally, SPOP can also be regulated by alcohol.^[^ ^61b^ ^]^ Alcohol intake has been shown to induce the expression of aldehyde dehydrogenase 2 (ALDH2) in colorectal cancer (CRC). Highly expressed ALDH2 can interact with the cytosolic domain of mPD‐L1 (260‐290) to inhibit the interaction between SPOP and PD‐L1 at K280, K281, and T290. This action can prevent polyubiquitination and enhance the stability of mPD‐L1. In addition to extensively investigated SPOP, several other E3 ligases are identified to possess the function of mPD‐L1 degradation as well. These E3 ligases include STUB1, ARIH1, Membrane‐associated RING‐CH 8 (MARCH8), RNF144A, TNF receptor associated factor 6 (TRAF6), ITCH and NEDD4. STUB1 can also regulate mPD‐L1 protein stability in cell membranes as well as its recycling endosomal by inducing polyubiquitination and lysosome‐dependent degradation. In this mechanism, STUB1 can indirectly or directly modify lysine residues in the cytoplasmic domain, leading to the destabilization of mPD‐L1.^[^ ^57a^ ^]^ When EGFR is suppressed or mutated, the mPD‐L1 is first phosphorylated by glycogen synthase kinase 3α (GSK3α). Next, ARIH1 can be recruited by the GSK3α phosphorylated mPD‐L1, leading to the ARIH1‐mediated K48‐linked polyubiquitination and proteasome‐mediated degradation.^[^ ^61a^ ^]^ The N‐terminal of MARCH8 is responsible for the degradation of multiple membranes and immune‐related proteins such as MHC‐II and DR4, which interacted with mPD‐L1 and induced ubiquitination and degradation of mPD‐L1 in NSCLC cells.^[^ ^61e^ ^]^ RNF144A, located in the cell membrane and acting as an E3 ubiquitin ligase, mainly promotes poly‐ubiquitination and degradation of fully glycosylated mPD‐L1.^[^ ^62^ ^]^ TRAF6, a type of plasma membrane adaptor protein that binds between the intracellular domains of cell surface and exists in a diverse range of organisms, positively regulates mPD‐L1 protein abundance through promoting the K63‐linked ubiquitination of PD‐L1.^[^ ^61f^ ^]^ ITCH promotes the internalization and lysosomal degradation of mPD‐L1 by controlling the ubiquitination of PD‐L1 K46 and K162 sites in a variety of cells.^[^ ^63^ ^]^ NEDD4 also serves as the ubiquitin enzyme for mPD‐L1 degradation in upper‐track carcinoma.^[^ ^61d^ ^]^ Mechanistically, increased fibroblast growth factor receptor 3 (FGFR3) can phosphorylate and activate NEDD4. Once activated, NEDD4 can interact with mPD‐L1 and catalyze Lys48 (K48)‐linked polyubiquitination, ultimately leading to the degradation of mPD‐L1. In addition, the ataxia telangiectasia and Rad3‐related (ATR) kinase inhibition also promotes the proteasomal degradation of mPD‐L1 protein. However, the specific ubiquitinase that mediates this process remains unclear.^[^ ^64^ ^]^

Besides ubiquitin‐mediated degradation, mPD‐L1 can also be downregulated by the HIP1R, potential surface metalloproteases (ADAM10/17), and autophagy factors such as p62. HIP1R can bind to mPD‐L1 with its 794–814 amino acid sequence, then the 966–976 sorting motifs shuttle them to the lysosome for further degradation.^[^ ^12^ ^]^ ADAM10/17 cleavage of mPD‐L1, resulting in the release of its N‐terminal fragment into the extracellular space, while the C‐terminal fragment is degraded by lysosomes. However, the specific mechanism remains unclear.^[^ ^65^ ^]^ Additionally, autophagy regulates PD‐L1 expression through p62/SQSTM1‐nuclear factor (NF)‐κB pathway.^[^ ^66^ ^]^

### 3.2 Therapeutic Strategies Targeting mPD‐L1 to Enhance Tumor Therapy

The expression regulation mechanisms described above provide potential therapeutic targets and agents for tumor treatment. The current therapies targeting mPD‐L1 can be categorized into two main fields: the direct strategies that directly inactivate or degrade mPD‐L1, and indirect strategies that target associated signaling pathways to indirectly modulate mPD‐L1. By inhibiting or degrading mPD‐L1, these approaches effectively elicit an antitumor immune response (**Table** **1**).

**Table 1:** Therapeutic agents for mPD‐L1 inhibition/degradation.

Agents	Target	Cancer type	The underlying mechanisms of PD‐L1 inhibition	Ref.
Atezolizumab, Avelumab, Durvalumab, Envafolimab	PD‐L1	Multiple tumors	mPD‐L1 and PD‐1/PD‐L1 pathway inhibition	[67, 73, 74, 75]
CA‐170	unknown	Advanced solid tumors and lymphomas	Unknown	[77a]
INCB086550, IMMH‐010, GS‐4224, MAX‐10181, ASC163, AN4005, BPI‐371153, Compound 17, S4‐1, BMS‐200, ZE132, YPD‐30, P39	PD‐L1	Multiple tumors	mPD‐L1 dimerization, thereby preventing mPD‐L1 from binding to PD‐1	[77, 78]
D‐Ap3–7c	PD‐L1	B16F10 cancer	preventing mPD‐L1 from binding to PD‐1	[79]
Compound P22, BMS‐37‐C3, Glue TAC, AbTACs, Ab‐3(LYTAC)	PD‐L1	Multiple tumors	mPD‐L1 ubiquitination and further degradation	[4, 80, 83, 84, 85]
Palbociclib, Abemaciclib	CDK4/6	Prostate cancer	mPD‐L1 degradation of by SPOP	[61c]
ES‐072, Osimertinib	EGFR	Breast cancer and NSCLC	mPD‐L1 degradation by ARIH1 and MARCH8	[61a,e]
VE‐822, AZD6738	ATR	MDA‐MB‐231 cells, A549 cells and HeLa cells	mPD‐L1 proteasomal degradation	[64]
Aspirin	HBXIP	Breast cancer	HBXIP inhibition to prevent mPD‐L1 acetylation by p300	[55]
2‐BP, PD‐PALM peptide	DHHC3	MC38 cells	DHHC3 inhibition to prevent mPD‐L1 palmitoylation, thus facilitating degradation	[56b]
PD‐LYLSO	HIP1R	HCT‐116, LOVO, and RKO cells	Accelerating mPD‐L1 transit to lysosomes and following degradation	[12]
Sunitinib	p62	B16F10 cells	mPD‐L1 autophagic degradation by p62	[86]
Ionomycin, PMAA	ADAM10/17	Human breast cancer cells	mPD‐L1 lysosomal degradation	[65]
Tubeimoside‐1	mTOR	LLC and B16 melanoma cells	mPD‐L1 lysosomal degradation	[87]
Metformin, Moroxydine	three positively charged juxtamembrane arginine residues	RKO cells	Destroying the electrostatic interaction between mPD‐L1 and acidic phospholipids, thus promoting mPD‐L1 ubiquitination and degradation	[29]
MCD, simvastatin	CRAC motifs of membrane	RKO cells	Inhibiting the binding of transmembrane domain of PD‐L1 to cholesterol	[58]
compound 6J1	Rab1	4T1 tumor and BI6F10 tumor	Downregulating mPD‐L1 by promoting exoPD‐L1 secretion	[88]

#### 3.2.1 The Direct Strategies

By activating the PD‐1/PD‐L1 signal pathways, the overexpressed mPD‐L1 in tumor cells can inactivate T cells, serving as a major barrier to the immune system's ability to eradicate tumors. Therefore, effective agents are needed to reverse this condition and activate immune cells into a “combat mode”. Classical mPD‐L1 agents include antibodies, small molecular inhibitors, and degraders, which can directly bind to mPD‐L1 to block the PD‐1/PD‐L1 signaling pathway. Antibodies and small molecular inhibitors can only bind to mPD‐L1, whereas degraders take a more comprehensive approach. In addition to binding to mPD‐L1, degraders hijack the cellular ubiquitination regulatory machinery to eliminate mPD‐L1, thus disrupting tumor immune tolerance and triggering a sustained immune response.

Concerning the category of antibodies, it is worth noting that antibodies targeting mPD‐L1 are key components of immunotherapy regimens and the only approved drugs currently used in the clinic. In 2016, the first anti‐PD‐L1 antibody, atezolizumab, was approved by Food and Drug Administration (FDA) for the treatment of metastatic/recurrent urothelial carcinoma.^[^ ^67^ ^]^ Clinical trials for urothelial carcinoma patients have gained satisfying outcomes.^[^ ^68^ ^]^ The study showed that 14.8% of patients experienced tumor shrinkage after atezolizumab treatment, and the therapeutic effect lasted longer than 13.8 months.^[^ ^69^ ^]^ In addition to urothelial carcinoma, atezolizumab has proven effective in treating NSCLC,^[^ ^70^ ^]^ breast cancer,^[^ ^71^ ^]^ and bladder cancer.^[^ ^72^ ^]^ Moreover, more anti‐PD‐L1 antibodies have become commercially available, including avelumab,^[^ ^73^ ^]^ durvalumab,^[^ ^74^ ^]^ and envafolimab (the first subcutaneous injection preparation).^[^ ^6^, ^75^ ^]^ These antibodies can inactivate mPD‐L1 and inhibit the PD‐1/PD‐L1 pathway, thus enhancing the killing effect of T cells toward tumor cells. However, poor response rates limit their clinical use, which is the reason why more effective and safer treatments are being sought.

Therefore, the development of new therapeutic agents to target mPD‐L1 is urgently needed. Compared with monoclonal antibodies, small‐molecular inhibitors possess advantages of small molecular weight and good oral bioavailability. Since the crystal structure of the human PD‐1/PD‐L1 complex was first published in 2015, several small‐molecule mPD‐L1 inhibitors have been reported.^[^ ^76^ ^]^ Bristol‐Myers Squibb (BMS) first reported a series of compounds with a resorcinol structure. Most of these small compounds could induce mPD‐L1 dimerization and then stabilize the mPD‐L1 homodimer or promote internalization into the cytosol, thereby preventing mPD‐L1 from binding to PD‐1, finally relieving the inhibition of PD‐L1‐dependent immunosuppressive effects. Since then, emerging small molecules from various corporations and other institutions have also entered clinical trials, including CA‐170 (Curis/Aurigene), INCB086550 (Incyte), IMMH‐010 (Chase Sun), GS‐4224 (Gilead), MAX‐10181 (Maxinovel), ASC163 (Ascletis), AN4005 (Adlai Nortye), and BPI‐371153 (BETTA).^[^ ^77^ ^]^ Additionally, some novel small molecules have been synthesized and have shown positive effects in promoting PD‐L1 degradation or inhibiting PD‐1/PD‐L1 interaction, such as Compound 17, S4‐1, BMS‐200, ZE132, YPD‐30, P39, and so on.^[^ ^76^, ^78^ ^]^ Further animal experiments demonstrated Compound 17 and ZE132 treatment can suppress tumor growth in vivo by promoting cytotoxic T‐cell tumor infiltration and activating antitumor immunity in the colon CT26 tumor model and melanoma B16‐F10 tumor model.^[^ ^76^, ^78^ ^]^ In addition, D‐Ap3‐7c, a type of bispecific aptamer which covalently conjugate to PD‐1 and mPD‐L1, can increase T‐cell infiltration and proliferation in the tumor region.^[^ ^79^ ^]^ These mPD‐L1 inhibitors are expected to avoid immunogenicity and improve public compliance and accessibility.

Also, proteolysis‐targeting chimera (PROTAC)^[^ ^4a^ ^]^ and lysosome‐targeting chimeras (LYTAC)^[^ ^80^ ^]^ provide a proof‐of‐concept approach for targeted mPD‐L1 degradation. These molecules often consist of an E3 ligase‐targeting moiety or a lysosome degradation tag and a PD‐L1 targeting moiety, connected by a linker. Upon entering tumor sites, one end (PD‐L1 ligand) selectively recognizes the mPD‐L1 protein, while the other binds to an E3 ubiquitin ligase or glucagon‐like peptide agonist, thereby hijacking the cellular ubiquitin‐proteasome or lysosomes pathway for mPD‐L1 degradation.^[^ ^81^ ^]^ Afterward, they can dissociate from the ternary complex for the next degradation cycle. Compared with small molecules, PROTAC and LYTAC offer a series of unique advantages, including low toxicity, high potency, selection, and abilities to target a broad range of diseases and overcome resistance. Currently, a few examples of PROTAC for PD‐L1 degradation have been reported. Cheng et al. synthesized a series of BMS‐based PROTACs with IC_50_ values ranging from 25 to 200 nm to achieve the degradation of mPD‐L1 in vitro and restore T cell proliferation, among these the most effective agents is compound P22.^[^ ^82^ ^]^ Yang et al. designed compound BMS‐37‐C3 to degrade mPD‐L1, thus enhancing T cell killing activity against melanoma.^[^ ^83^ ^]^ Wang et al. synthesized a dual specificity antibodies that can recruit RNF43 (AbTACs, an E3 ubiquitin ligase located in the cell membrane) to mPD‐L1, thus promoting the ubiquitination and subsequent degradation of proteins.^[^ ^84^ ^]^ Zhang et al. developed a covalently engineered nanobody chimeras (Glue TAC) for mPD‐L1 lysosomal degradation, thus inhibiting the tumor growth significantly.^[^ ^85^ ^]^ Banik et al. reported a type of LYTAC (Ab‐3), which could recruit mPD‐L1 to lysosomes for degradation.^[^ ^80^ ^]^ By degrading mPD‐L1, both PROTAC and LYTAC help unleash adaptive immune response, showing the potential for cancer treatment. However, the studies on PROTAC and LYTAC for mPD‐L1 degradation are at an initial stage of investigation and far from clinical practice. Further exploration is still needed to evaluate the long‐term safety and potential side effects after fully PD‐L1 degradation, providing the theoretical basis for further clinical application of PROTAC and LYTAC.

#### 3.2.2 The Indirect Strategies

Besides directly inactivating or degrading mPD‐L1, the expression levels of mPD‐L1 are also indirectly downregulated by other signaling pathways. As has been referred to above, the concentration of ubiquitination, acetylation/deacetylation, and palmitoylation can regulate the level of mPD‐L1. Several therapeutic agents have been shown to regulate these associated signaling pathways to eventually downregulate mPD‐L1 expression to perform as a new type of immune therapy for tumor patients.

Generally, activating the ubiquitination process of mPD‐L1 can promote its degradation,^[^ ^89^ ^]^ thus activating anti‐tumor responses. By increasing E3 ubiquitin ligase activity such as SPOP, STUB1, ARIH1, and MARCH8, the degradation of mPD‐L1 can be facilitated, resulting in T cell activation and increased anti‐tumor responses. CDK4/6 inhibitors, such as palbociclib and abemaciclib, can promote the recognition and degradation of mPD‐L1 by SPOP. The combination of CDK4/6 inhibitor treatment and PD‐L1 antibodies can enhance tumor regression.^[^ ^61c^ ^]^ H1A antibody can inhibit the interaction of mPD‐L1 with CMTM6, a membrane‐stabilizing the protein, thus promoting polyubiquitination and degradation of mPD‐L1 by STUB1 and further regulating anti‐tumor immunity.^[^ ^57b^ ^]^ EGFR inhibitors, such as ES‐072 and osimertinib, can induce both ARIH1‐ and MARCH8‐mediated degradation and showed a marked suppression in breast cancer and NSCLC.^[^ ^61a,e^ ^]^ ATR inhibitors, including VE‐822 and AZD6738 promote the proteasomal degradation of PD‐L1 through an unknown ubiquitinase pathway.^[^ ^64^ ^]^

Other post‐translational modifications, including acetylation/deacetylation and palmitoylation, can also regulate mPD‐L1 expression. Several small‐molecular agents acting on these sites can enhance the antitumor function of the immune system. As stated in Section 3.1.1, acetyltransferase p300 is involved in the acetylation modification of mPD‐L1, allowing mPD‐L1 protein to reside in cell membranes. Thus, inhibiting p300 can lead to a reduction of mPD‐L1. HBXIP can induce acetylation modification of mPD‐L1 through p300, and aspirin can inhibit HBXIP to prevent mPD‐L1 acetylation by p300, leading to tumor growth suppression in breast cancer.^[^ ^55^ ^]^ Additionally, palmitoyltransferase DHHC3 catalyzes the palmitoylation of mPD‐L1, which stabilizes mPD‐L1 by inhibiting ubiquitination. Inhibition of DHHC3 by 2‐BP and PD‐PALM peptide can inhibit mPD‐L1 expression in mice bearing MC38 cells, thus enhancing T‐cell immune responses against the tumor.^[^ ^56b^ ^]^

Other therapeutic agents for downregulating mPD‐L1 expression, including peptides, autophagy regulators, and some other small molecules, have been explored to enhance the anticancer immune response. HIP1R can bind and deliver mPD‐L1 to lysosomes for degradation. A new peptide, PD‐LYLSO, has been developed from the binding sequences and sorting sequences of HIP1R to accelerated mPD‐L1 transit to lysosomes and following degradation.^[^ ^12^ ^]^ p62 promotes mPD‐L1 translocation into autophagic lysosomes for degradation, and sunitinib promotes p62 accumulation, thereby promoting p62‐mediated autophagic degradation of mPD‐L1 and generating an antitumor immune response, and subsequently extending survival in the B16F10 mice.^[^ ^86^ ^]^ Ionomycin and PMAA, the activators of ADAM10/17, can enhance the lysosomal degradation of mPD‐L1.^[^ ^65^ ^]^ Tubeimoside‐1 effectively targets the mechanistic target of mTOR and subsequently triggers the degradation of mPD‐L1, thereby ultimately fostering the enhancement of antitumor immune responses.^[^ ^87^ ^]^ mPD‐L1 stabilizes itself by binding to the acidic phospholipids, while metformin and moroxydine can destroy the electrostatic interaction between mPD‐L1 with the acidic phospholipids, thus enhancing ubiquitination and degradation of mPD‐L1 proteins.^[^ ^29^ ^]^ mPD‐L1 also binds to the membrane through its transmembrane domain binding to cholesterol, and MCD or simvastatin treatment can down‐regulate mPD‐L1.^[^ ^58^ ^]^ Additionally, a triazine compound 6J1 can downregulate mPD‐L1 by promoting exoPD‐L1 secretion, which can increase the secretion of some chemokines to facilitate the recruitment of tumor‐infiltrating CD8^+^ T cells to tumor microenvironments, thus significantly promoting the anticancer immune response.^[^ ^88^ ^]^ These approaches can enhance the antitumor function of the immune system and the body's natural ability to combat cancer, providing promising strategies for cancer treatment.

## 4 cPD‐L1 Expression Regulation Mechanisms and Therapeutic Strategies for Tumor Therapy

**Figure 5:**
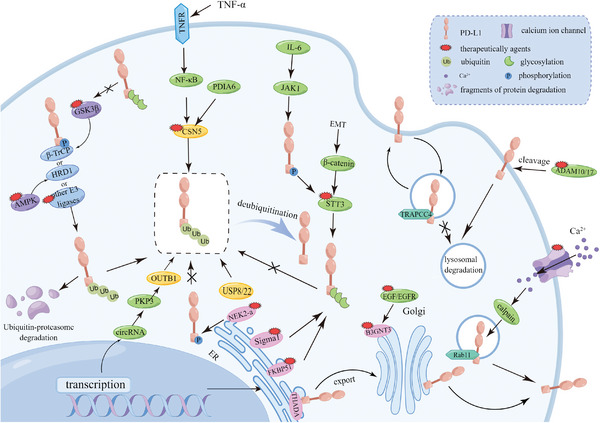
cPD‐L1 expression regulation mechanisms and therapeutic strategies. The body can regulate the content of cPD‐L1 through various signaling pathways. cPD‐L1 downregulation is primarily mediated by ubiquitination‐proteasome pathway and blue ovals represent E3 ubiquitination ligases. cPD‐L1 upregulation mechanisms include inhibition of E3 ubiquitination ligases (purple ovals represent kinases which can inhibit the activity of E3 ubiquitination ligases), removal of ubiquitin chain (yellow ovals represent deubiquitylating enzymes), and stabilization modification of cPD‐L1 (pink ovals represent glycosylation modifying enzymes which can protect cPD‐L1 from ubiquitination modification). The small red polygons denote small molecular modulators targeting the corresponding targets.

In this section, we conclude the impact of posttranslational modifications on cPD‐L1 stability, encompassing glycosylation, phosphorylation, ubiquitination, and deubiquitination. Naturally, other pathways exist to regulate cPD‐L1 levels. Similar to our earlier overview of mPD‐L1, we have also classified cPD‐L1 regulation mechanisms based on their upregulating or downregulating effects (**Figure** **5**). Furthermore, we provide a summary of compounds with potential activity against cPD‐L1. Although researches in this area are still in its infancy, investigators are actively exploring these agents for their potential to eliminate tumors.

### 4.1 Regulation of cPD‐L1 Expression

A range of regulatory mechanisms in the cell can regulate the content of cPD‐L1. There are two main sources of cPD‐L1. One is newly synthesized PD‐L1 in ER and Golgi. Newly synthesized PD‐L1 undergoes various modifications in the ER and Golgi to form matured PD‐L1, and then plays a role in the cytoplasm or is translocated to the membrane. The other is recycled back mPD‐L1 from the membrane. mPD‐L1 is internalized and recycled to form cPD‐L1 or nPD‐L1. In the following, we give a specified introduction to these mechanisms and related studies.

#### 4.1.1 Mechanisms for Up‐Regulation of cPD‐L1

cPD‐L1 can be targeted for degradation by E3 ligase, however, ubiquitinated cPD‐L1 can also be reversed by deubiquitinases or deubiquitylating enzymes (DUBs), which cleave and remove the ubiquitin chains from cPD‐L1 to protect it from degradation. Moreover, several post‐translational modifications, including glycosylation and phosphorylation, and other pathways are used to protect cPD‐L1 from lysosomal degradation. A more detailed description of these pathways will be given later.

Numerous deubiquitinating enzymes have been identified, including COP9 signalosome 5 (CSN5), otubain‐1 (OTUB1), and USP22. CSN5 is one of the most extensively studied deubiquitinases.^[^ ^90^ ^]^ Mechanistically, K48‐linked ubiquitin chains are canonical signals for cPD‐L1 degradation, and CSN5 removes K48‐linked ubiquitin chains and then stabilizes cPD‐L1 expression. Therefore, upregulation of CSN5 expression can prevent the degradation of cPD‐L1. On the one hand, TNF‐α secretion by M2 macrophages induces the expression of CSN5 through activation of the NF‐κB, which subsequently leads to deubiquitination of cPD‐L1 and then blocks the degradation. On the other hand, Protein Disulfide Isomerase Family A Member 6 (PDIA6), an ER resident protein involved in the protein folding and disulfide bond formation, also promotes the formation of CSN5 disulfide bonds, which in turn results in inhibition of PD‐L1.^[^ ^91^ ^]^ OTUB1, another deubiquitinating enzyme that belongs to the OTU family of cysteine proteases 4, is also highly specific for cleaving K48‐linked polyubiquitin chains.^[^ ^92^ ^]^ It can remove the Lys‐48‐type polyubiquitination chain of cPD‐L1, thus inhibiting ER‐mediated degradation.^[^ ^92^ ^]^ Moreover, increased circular RNAs (circRNA) are associated with upregulation of OUTB1. This, in turn, promotes the stabilization of cPD‐L1 through OUTB1's deubiquitination activity.^[^ ^93^ ^]^ USP22 can also directly or indirectly stabilize cPD‐L1 through its deubiquitination activity.^[^ ^94^ ^]^ It removes K6, K11, K27, K29, K33 and K63‐linked ubiquitin chains of cPD‐L1, and, in combination with CSN5 to promote cPD‐L1 stability.^[^ ^94a^ ^]^ Moreover, USP22 also contributes to immune escape by reducing the degradation of SPI1 protein, which enhances PD‐L1 transcription.^[^ ^94b^ ^]^ The deubiquitylase activity of USP8 is also required for stabilizing PD‐L1.^[^ ^95^ ^]^ USP8 specifically interacts with cPD‐L1 to remove its K63‐linked poly‐ubiquitination, which results in the stability of PD‐L1 and arrests its degradation.^[^ ^61f^ ^]^

The stability of cPD‐L1 can be enhanced by glycosylation modification, which can impede its degradation. It is a process by which the glycan chains are added to a protein, catalyzed by glycosyltransferase enzymes.^[^ ^96^ ^]^ N‐glycosylation plays a role in cPD‐L1's biosynthesis, stability, and subcellular localization.^[^ ^9c^ ^]^ N‐glycosylation prevents cPD‐L1 degradation through the GSK3β‐mediated 26S proteasome.^[^ ^4b^ ^]^ The major N‐glycosylation sites of PD‐L1 reside at N35, N192 (N191 in mouse PD‐L1), N200 (N199 in mouse PD‐L1), and N219 (N218 in mouse PD‐L1).^[^ ^97^ ^]^ Various enzymes catalyze the glycosylation of cPD‐L1, including β−1,3‐N‐acetylglucosaminyl transferase (B3GNT3), STT3, FK506 binding protein 51 (FKBP51) and Sigma1. B3GNT3 is a glycosylase enzyme that distributes in Golgi and catalyzes the attachment of poly‐N‐acetyllactosamines at N192 and N200 sites on cPD‐L1. In tumor cells, high expression of epidermal growth factor (EGF)/EGFR activates B3GNT3, leading to cPD‐L1 glycosylation.^[^ ^97^, ^98^ ^]^ STT3 is an ER N‐glycosyl transferase that catalyzes the first N‐linked glycosylation.^[^ ^99^ ^]^ On the one hand, STT3 is critical for epithelial–mesenchymal transition (EMT)‐mediated cPD‐L1 protein induction. Specifically, the EMT process increases the expression of N‐glycosyltransferase STT3 through β‐catenin, and STT3 catalyzes the glycosylation of 4 sites, subsequently inducing cPD‐L1 N‐linked glycosylation to stabilize and upregulate cPD‐L1 in cancer stem‐like cells. On the other hand, IL‐6/JAK1 can also activate STT3, resulting in the glycosylation of cPD‐L1.^[^ ^100^ ^]^ FKBP51 serves as a chaperone within the ER lumen to assist in cPD‐L1 protein folding and glycosylation, thus stabilizing cPD‐L1 expression.^[^ ^101^ ^]^ Sigma1 can regulate glycosylation pathways of cPD‐L1 in ER, thereby preventing autophagic degradation of cPD‐L1.^[^ ^102^ ^]^ However, FKBP51‐ and Sigma1‐mediated glycosylation regulation of PD‐L1 needs further elucidation.

Phosphorylation can change the activity and stability of cPD‐L1 rapidly. It describes the addition of the phosphate group to proteins. Studies have uncovered differential biological processes and effects of PD‐L1 phosphorylation at different sites. The positive regulation of cPD‐L1 is supported by the following examples. Several phosphorylation enzymes, including GSK3β, Janus kinase‐1 (JAK1), never in mitosis gene a‐related kinase 2 (NEK2) and casein kinase 2 (CK2), can phosphorylate cPD‐L1 and then protect it from proteasomal degradation. GSK3β can phosphorylate PD‐L1 at T180 and S184 sites, resulting in phosphorylation‐dependent proteasome degradation of cPD‐L1 by β‐transducin repeat containing E3 ubiquitin‐protein ligase (β‐TrCP) mediated ubiquitination. JAK1 can phosphorylate cPD‐L1 at Y112 to recruit N‐glycosyltransferase STT3A and then cause cPD‐L1 glycosylation modification.^[^ ^100^ ^]^ Also, NEK2, a cell cycle‐regulated protein, can phosphorylate cPD‐L1 inside the ER lumen at T193/T209, thereby preventing ubiquitination‐mediated proteasome degradation.^[^ ^103^ ^]^ CK2, a well‐known protein kinase, phosphorylates cPD‐L1 at Thr285 and Thr290, disrupting its binding with cullin 3 and protecting cPD‐L1 from cullin 3‐mediated proteasomal degradation.^[^ ^104^ ^]^

Besides previously discussed three primary regulatory mechanisms, cPD‐L1 can defend itself against lysosomal degradation through several strategies. First, cPD‐L1 expression can be regulated by Golgi‐related regulators, as further processing in the Golgi contributes to its maturation. The thyroid adenoma associated gene (THADA) maintains the residency of cPD‐L1 in the Golgi, thus promoting its maturation and facilitating intracellular transport.^[^ ^105^ ^]^ Mechanistically, THADA fastens cPD‐L1 to COPII vesicles by Sec24A‐mediated anchoring, thereby enabling cPD‐L1 transport from ER to the Golgi and stabilizing its Golgi residency. Second, cPD‐L1 can form a stable conformation by binding to TRAPPC4, a type of vesicular trafficking protein.^[^ ^13^ ^]^ Specifically, TRAPPC4 vesicles stabilize cPD‐L1 in Rab11‐positive recycling endosomes, preventing cPD‐L1 from degradation in lysosomes. Lastly, the expression of cPD‐L1 can be augmented by high intracellular calcium levels, which activate calpain and promote the stabilization of cPD‐L1.^[^ ^106^ ^]^

#### 4.1.2 Mechanisms for Down‐Regulation of cPD‐L1

The down‐regulated mechanism of cPD‐L1 primarily involves the degradation by the proteasome. cPD‐L1 can be ubiquitinated by specific E3 ubiquitin ligases and degraded by proteases or lysosomes. These E3 ubiquitin ligases include β‐TrCP, HMG‐CoA reductase degradation 1 (HRD1), FBXO22 and ring finger protein 125 (RNF125). β‐TrCP and HRD1 can ubiquitinate phosphorylated cPD‐L1. In the β‐TrCP‐mediated process, cPD‐L1 is phosphorylated by GSK3β at T108 and S184 sites, which in turn ubiquitinated by β‐TrCP and degraded by proteasome.^[^ ^4b^ ^]^ In the HRD1‐mediated process, cPD‐L1 is phosphorylated at the S195 site by activated AMPK and then abnormally glycosylated. This dysregulated cPD‐L1 then undergoes HRD1‐induced polyubiquitination and ER‐mediated degradation.^[^ ^107^ ^]^ FBXO22, a member of the F‐box protein family, has been proved to be an E3 ligase and degrades cPD‐L1 through ubiquitination and proteasome,^[^ ^108^ ^]^ but the detailed mechanism is less clear. Beyond these, E3 ubiquitin ligase RNF125 also mediates the degradation of cPD‐L1 in HNSCC.^[^ ^109^ ^]^ However, the mechanistic roles of RNF125 in directing action site‐specific ubiquitination of cPD‐L1 are not understood at the molecular level.

### 4.2 Therapeutic Strategies Targeting cPD‐L1 to Enhance Tumor Therapy

cPD‐L1 mainly originates from two sources: one arises from recycled mPD‐L1, and the other originates from new‐synthesized PD‐L1 in the cytoplasm.^[^ ^57b^ ^]^ The efforts to deplete or degrade cPD‐L1 or “recycle” mPD‐L1 not only inhibit pro‐oncogenic signaling but also block the cPD‐L1 protein transport to the cell membrane. Therefore, cPD‐L1 can be a more effective therapeutic target, which would be expected to produce greater inhibition over a wide range, thus promoting the activation of T cells. Inhibitors for cPD‐L1 are a new field of immunotherapy, and several researchers have made their answers. It is worth noting that most strategies regulate cPD‐L1 content by modulating associated signaling pathways, involving ubiquitination/deubiquitination, glycosylation, and phosphorylation regulation, thus impacting tumor immune response (**Table** **2**).

**Table 2:** Therapeutic agents for cPD‐L1 inhibition/degradation.

Agents	Target	Cancer type	The underlying mechanisms of PD‐L1 inhibition	Ref.
Metformin	HRD1	Breast cancer	cPD‐L1 degradation via HRD1	[110]
Gefitinib, erlotinib, lapatinib, AG1478	EGFR	Multiply tumors	Inactivation GSK‐3β, thereby suppressing cPD‐L1 glycosylation and promoting degradation by β‐TrCP	[4b]
Roscovitine	FBXO22	NSCLC	cPD‐L1 ubiquitination and degradation by FBXO22	[108]
Curcumin, berberine, shikonin	CSN5	Basal‐like breast cancer, NSCLC, and Pancreatic cancer respectively	Inhibiting the deubiquitination activity of CSN5, thus diminish cPD‐L1	[90, 111, 112]
DUBs‐IN‐2	UPS8	MC38 tumor	cPD‐L1 degradation by USP8	[61f]
Etoposide	EMT	Cancer stem‐like cells and Lewis lung cancer	hindering cPD‐L1 glycosylation and promoting its degradation	[99, 114]
Gefitinib	EGFR	Lung adenocarcinoma	decreasing cPD‐L1 glycosylation and increasing degradation	[98]
SAFit	FKBP51s	Glioma	PD‐L1 degradation by inhibiting glycosylation	[101]
Resveratrol	gly‐PD‐L1‐processing enzymes and GSK‐3β	Breast carcinomas, lung cancer	Inducing abnormal glycosylation and dimerization of cPD‐L1; inducing phosphorylation of cPD‐L1 and its proteasomal degradation	[113, 115]
NCL00017509	NEK2	Pancreatic ductal adenocarcinoma	inhibition cPD‐L1 phosphorylation and its degradation	[103]
BMS1166	PD‐L1	Human lung cancer PC‐9 cell line	Blocking the transporting of cPD‐L1 from ER to Golgi apparatus and its further proteasomal degradation	[116]
Verteporfin	Autophagy	Ovarian, osteoblastoma, and lung cancers	Inducing the autophagy of cPD‐L1 and its degradation	[117]
SA‐49	PKCα	NSCLC	Inducing PKCα activation and subsequent GSK3β suppression, resulting in cPD‐L1 degradation	[118]
9‐ING‐41	GSK‐3β	T‐cell leukemia/lymphoma	Inhibiting of GSK‐3β to suppress cPD‐L1 expression	[119]
Lercanidipine	STAT1	NCI‐H1299	Increasing phosphorylation of cPD‐L1 by STAT1, further resulting in its degradation	[120a]
Amlodipine, Lercanidipine, Compound F4	Calcium channel	RKO cells, MDA‐MB‐231 cells, H1299 cells and H292 cells	Reducing the level of cytosolic calcium ion and cleavage of Becklin1 by Calpain, thus decreasing cPD‐L1 expression	[106, 120]
Ionomycin	ADAM10/17	Breast cancer	Promoting cPD‐L1 cleavage of metalloproteinases ADAM10/17, thus leading to cPD‐L1 lysosomal degradation	[65]

The content of cPD‐L1 is regulated by ubiquitination/deubiquitination enzymatic activity. On the one hand, increased E3 ligase activities, including HRD1, β‐TrCP, and FBXO22, have been shown to promote cPD‐L1 ubiquitination and degradation. Metformin has been reported to degrade cPD‐L1 via HRD1‐mediated degradation.^[^ ^110^ ^]^ Gefitinib, erlotinib, lapatinib, or AG1478 destabilize PD‐L1 and enhance β‐TrCP‐mediated degradation, thus potentiating the efficacy of PD‐L1 antibodies.^[^ ^4b^ ^]^ Roscovitine increases the expression of FBXO22, which can, in turn, decrease cPD‐L1, and the combination of roscovitine and immune checkpoint inhibitors as a strategy can enhance immune responses.^[^ ^108^ ^]^ On the other hand, the inhibition of cPD‐L1 deubiquitinases activity, including CSN5 and USP8, can prevent ubiquitinated cPD‐L1 from reverting to non‐ubiquitinated cPD‐L1, then facilitating the degradation of ubiquitinated cPD‐L1. Curcumin,^[^ ^90^ ^]^ berberine,^[^ ^111^ ^]^ and shikonin^[^ ^112^ ^]^ diminish cancer cell cPD‐L1 expression via inhibiting the deubiquitination activity of CSN5, thus reversing immune escape and facilitating antitumor immunity in breast cancer and pancreatic cancer. And DUBs‐IN‐2, a UPS8 inhibitor, retains cPD‐L1 ubiquitination by promoting USP8 cytoplasmic degradation, thus promoting cPD‐L1 degradation and facilitating antitumor immunity in MC38 tumor model.^[^ ^61f^ ^]^

Glycosylation can also regulate the stability of cPD‐L1 (as described in Section 4.1.1), and the inhibition of cPD‐L1 glycosylation can facilitate its degradation. First, the inhibition of N‐glycosylation is also associated with anti‐tumor effects. Resveratrol has been shown to inhibit the activity of gly‐PD‐L1‐processing enzymes, which modulate the N‐linked glycan decoration of cPD‐L1, thereby inducing abnormal glycosylation and dimerization of cPD‐L1.^[^ ^113^ ^]^ Second, inhibiting cPD‐L1 glycosylase activity, including STT3, B3GNT3, and FKBP51s, have been confirmed as valid strategies. Etoposide suppresses STT3 activity through EMT inhibiting, thus hindering cPD‐L1 glycosylation and promoting its degradation, ultimately inducing tumor‐specific immunity.^[^ ^99^, ^114^ ^]^ EGFR inhibitor gefitinib restrains B3GNT3 activity, leading to decreased cPD‐L1 glycosylation and increased degradation, which in turn provides a new insight to increase the efficacy of immunotherapy in lung adenocarcinoma patients.^[^ ^98^ ^]^ SAFit is shown to facilitate PD‐L1 degradation by inhibiting FKBP51s‐mediated glycosylation in glioma.^[^ ^101^ ^]^

Phosphorylation at specific sites also upregulates cPD‐L1 expression in the cytosol. Because phosphorylation is dependent on phosphorylation kinases, one viable alternative approach is to inhibit kinase activity, including GSK3β and AMPK. In addition to inhibiting gly‐PD‐L1‐processing enzymes, resveratrol can also activate GSK3β and then phosphorylate cPD‐L1, leading to its proteasomal degradation.^[^ ^115^ ^]^ Metformin activates AMPK and phosphorylates cPD‐L1 at S195, leading to its HRD1‐mediated degradation. Notably, breast cancer patients treated with metformin exhibit lower cPD‐L1 expression, and combined treatment with a CTLA‐4 antibody effectively inhibited tumor growth.^[^ ^110^ ^]^ NCL00017509, a type of NEK2 inhibitor, can inhibit cPD‐L1 phosphorylation at T194/T210, resulting in an unstable cPD‐L1 conformation and its degradation.^[^ ^103^ ^]^ In addition, the expression level of cPD‐L1 is also regulated by other regulatory mechanisms, which influence anti‐tumor immunity. BMS1166 impedes the transporting of cPD‐L1 from ER to Golgi and its further glycosylation, leading to its degradation through proteasomes, and eventually relieving immune suppression and activating T cells.^[^ ^116^ ^]^ Verteporfin induces the autophagy of cPD‐L1 in the Golgi‐associated autophagy pathway and then exerts antitumor efficacy.^[^ ^117^ ^]^ SA‐49‐induces MITF translocation acts through the activation of PKCα and subsequent suppression of GSK3β activity and degradation of PD‐L1. In further in vivo experiments, SA‐49 suppresses the progression of xenograft tumors by activating the immune microenvironment.^[^ ^118^ ^]^ 9‐ING‐41 selectively inhibits GSK‐3β, thus suppressing cPD‐L1 expression and subsequently exerting excellent in vivo antitumor activity against refractory adult T‐cell leukemia/lymphoma.^[^ ^119^ ^]^ Calcium channel blockers, including amlodipine, lercanidipine, and compound F4, can decrease cPD‐L1 expression by reducing the level of cytosolic calcium ion and cleavage of Becklin1 by Calpain, which can improve the efficacy of anti‐PD‐L1 antibodies.^[^ ^106^, ^120^ ^]^ Lercanidipine also increases the phosphorylation of STAT1 at Tyr701 and Ser727, inducing the degradation of cPD‐L1.^[^ ^120a^ ^]^ Ionomycin enhances the lysosomal degradation of cPD‐L1 by promoting cPD‐L1 cleavage of metalloproteinases ADAM10/17.^[^ ^65^ ^]^

Overall, the modulation of cPD‐L1 levels through the regulation of related signaling pathways holds great potential for achieving a curative intent. However, some questions deserve further attention. First, these strategies frequently target other sites before cPD‐L1 degradation occurs, which may lead to a delayed therapeutic effect compared to direct strategies. Second, compensatory mechanisms that lead to compensatory activation of other pathways may exist. As a result, some agents may not exert their therapeutic effects in vivo. As such, more in vivo studies are needed to rigorously verify these results.

## 5 exoPD‐L1 Expression Regulation Mechanisms and Therapeutic Strategies for Tumor Therapy

### 5.1 Regulation of exoPD‐L1 Expression

A steady stream of exoPD‐L1 is secreted and plays a critical role in tumor escape. On the one hand, exoPD‐L1 acts on the immediately adjacent cancer cells and macrophage cells by paracrine signals, facilitating PD‐L1 expression in adjacent tumor cells and M2 polarization in adjacent macrophage cells, and finally inducing immune evasion.^[^ ^49^ ^]^ On the other hand, they can enter into circulation, spread distantly to the lymph nodes, and exert suppressive effects on a newly generated T cell that can express PD‐1. Therefore, a better understanding of the regulation of exoPD‐L1 expression in tumor cells is essential for cancer therapy. It is worth noting that few studies focused on the exoPD‐L1, so we only summarize the upregulation mechanisms.

**Figure 6:**
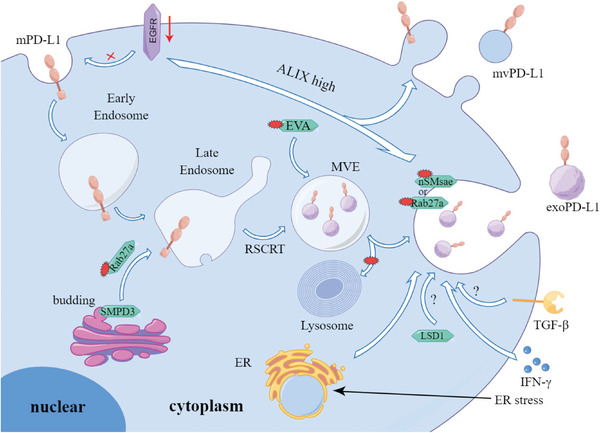
ExoPD‐L1 expression regulation mechanisms and therapeutic strategies for tumor therapy. ExoPD‐L1 can be derived not only from mPDL1 but also from the newly synthesized cPD‐L1 in Golgi apparatus. Moreover, some cytokines, such as transforming growth factor β (TGF‐β) and IFN‐γ, can also promote exoPD‐L1 production. The green polygons represent the enzymes involved in regulation exoPD‐L1, and the small red polygons denote small molecular modulators targeting the corresponding targets.

Understanding how exoPD‐L1 is regulated has led to a detailed mechanistic dissection of the production of exoPD‐L1 (**Figure** **6**). Research has demonstrated that exoPD‐L1, which originates from the plasma membrane, shares a comparable topology to mPD‐L1.^[^ ^14^, ^38^ ^]^ During this process, mPD‐L1 is internalized into endosomes by endocytosis, then forms multivesicular endosomes (MVE) and finally gives rise to exoPD‐L1. During the stages of exosome biosynthesis and secretion, SMPD3 initiates vesicle budding, Rab27 oversees the fusion of multivesicular bodies (MVBs) with the plasma membrane, the endosomal sorting complex required for transport (ESCRT) manages MVE maturation, and nSMase2 and Rab27 regulate exosome secretion. The expression level of exoPD‐L1 is determined by two main factors: enzymes involved in exosome formation and feedback regulation mechanisms originating from exoPD‐L1 within the exosome. Throughout the steps of exosome biosynthesis and secretion, several key molecules play crucial roles. SMPD3 initiates vesicle budding, Rab27 oversees the fusion of MVE with the plasma membrane, the endosomal sorting complex required for transport (ESCRT) manages MVE maturation, and neutral sphingomyelinase type 2 (nSMase2) and Rab27 regulate exosome secretion.^[^ ^121^ ^]^ The process begins with tumor cell membranes forming endosomes that translocate mPD‐L1 into the cytoplasm via the clathrin‐mediated endocytic pathway. These endosomes mature into MVE with the involvement of endothelin receptor A (ETA), although the specific molecular mechanisms of this regulation remain unclear and secreted. MVE then secretes exoPD‐L1 into extracellular space, a process dependent on Rab5 and Rab27.^[^ ^88^, ^122^ ^]^ Additionally, ALG‐2 interacting protein X (ALIX) can also modulate exosome content and control the balance between exoPD‐L1 and mPD‐L1.^[^ ^123^ ^]^ More specifically, the high expression of ALIX results in the increase of both exoPD‐L1 and mvPD‐L1, and a decrease of mPD‐L1, and vice versa. Furthermore, histone lysine‐specific demethylase 1 (LSD1) promotes the secretion of exoPD‐L1, and increased activity of these enzymes may positively impact exoPD‐L1 content.^[^ ^124^ ^]^ Except for tumor cells, dendritic cells and macrophages can also release exoPD‐L1, but the exact mechanisms underlying this process have yet to be fully elucidated.

Alternatively, some cytokines, such as transforming growth factor β (TGF‐β) and IFN‐γ, have also been found to promote exoPD‐L1 production. TGF‐β promotes the production of exoPD‐L1 in a dose‐dependent manner in breast cancer cells,^[^ ^125^ ^]^ and IFN‐γ also stimulates the levels of exoPD‐L1 in melanoma cells and salivary adenoid cystic carcinoma.^[^ ^14^, ^126^ ^]^ Besides, ER stress and smoking also induce the release of exoPD‐L1 from HNSCC and NSCLC.^[^ ^49^, ^127^ ^]^ However, details on these events are also not provided.

### 5.2 Therapeutic Strategies Targeting exoPD‐L1 to Enhance Tumor Therapy

The release of exoPD‐L1 by tumor cells into the extracellular milieu leads to its transport to remote lymph nodes, where it suppresses T cells and facilitates immune escape. Inhibition of exosome secretion could potentially restore the anti‐tumor T cell response by eliminating T cell inhibition. Increasing studies are becoming focused on exoPD‐L1, so it is expected to become a new target for future cancer treatment. However, the development of effective therapeutic agents for exoPD‐L1 is still in its infancy and no therapeutic strategies directly targeting exoPD‐L1 are available. The existing therapeutic agents almost exclusively use exosome inhibitors to suppress the production of the exosome, in turn, repress production of exoPD‐L1. More specific descriptions of these agents are as follows (Figure 6).

Several agents target different aspects of exosome production, secretion, and maturation to inhibit exoPD‐L1 and improve anti‐cancer immunity (**Table** **3**). First, some agents act on the specific enzyme, such as N‐SMase2 and Rab27a, that are involved in the incidence of exosome production and secretion, thus inhibiting exoPD‐L1. GW4869 can decrease exosome release by inhibiting N‐SMase2, thus decreasing exoPD‐L1 secretion.^[^ ^128^ ^]^ Atorvastatin suppresses exosome production by inhibiting Rab27a.^[^ ^129^ ^]^ Second, ETA inhibitors, including sulfisoxazole, bosentan, ambrisentan, and macitentan, act on MVE maturation and inhibit the secretion of exoPD‐L1, thereby improving anti‐cancer immunity.^[^ ^122^, ^130^ ^]^ When used in conjunction with PD‐L1 antibodies, sulfisoxazole and macitentan have been shown to significantly reduce exoPD‐L1 levels in the plasma of a breast cancer mouse model, activate cytotoxic T cells, and suppress tumor growth and metastasis.^[^ ^130^ ^]^ Third, some other agents, including temsirolimus, rapamycin, and everolimus, downregulate exoPD‐L1 levels by inducing autophagy.^[^ ^131^ ^]^ It has been extensively shown that autophagy activation inhibits exosome secretion by promoting the co‐localization of MVB with autophagosome.^[^ ^132^ ^]^ In addition, α‐helical (AH‐D) peptide can inhibit various tumor derived exosomes by targeting the phospholipid bilayer of exosomes membranes, thus disrupting exoPD‐L1. And the combination treatment of AH‐D peptide and PD‐1 antibodies can effectively inhibit the growth of the tumor.^[^ ^133^ ^]^ They can also enhance the efficiency of anti‐PD‐L1 therapy by regulating Rab, leading to the degradation of exoPD‐L1.^[^ ^131^ ^]^

**Table 3:** Therapeutic agents for exoPD‐L1 inhibition/degradation.

Agents	Target	Cancer type	The underlying mechanisms of PD‐L1 inhibition	Ref.
GW4869	N‐SMase2	B16F10 cancer	Inhibiting N‐SMase2 to decrease exoPD‐L1 secretion	[128]
Atorvastatin	Rab27a	Breast cancer	Suppression exosome production	[129]
Sulfisoxazole, bosentan, ambrisentan, macitentan	ETA	CT26, Breast cancer	Acting on MVE maturation and inhibiting exoPD‐L1 secretion	[122, 130]
Temsirolimus, rapamycin, everolimus	Rab	Breast cancer	Upregulating Rab and inducing autophagy, leading to exoPD‐L1 degradation	[131]
AH‐D peptide	Exosome membrane	B16F10 cancer	Disrupting membrane of exosome, thus decreasing exoPD‐L1	[133]

In conclusion, these agents provide potential therapeutic strategies for targeting exoPD‐L1 and enhancing anti‐cancer immunity. By inhibiting specific enzymes, MVE maturation, or autophagy, these agents work to reduce exosome production and secretion, leading to a decrease in exoPD‐L1 and an improvement in anti‐cancer immune responses. Moreover, the regulation of exoPD‐L1 expression is a complex process involving multiple factors and signaling pathways. Current therapeutic strategies targeting exoPD‐L1 show promising results in preclinical studies and have the potential to become a new direction for cancer treatment. However, further research is needed to address the challenges associated with these strategies and to explore their clinical efficacy in combination with other therapies.

## 6 Conclusion and Future Perspective

Immunotherapy is a booming field bound to change cancer therapy. Compared with the pyrrhic victory of classical therapy (chemotherapy or radiotherapy), it leverages the human immune system to fight cancer, akin to recovering from a cold and avoids the toxic side effects of harsh chemicals and radiation. Immune checkpoints, such as PD‐L1, may hold the key to unlocking this potential. PD‐L1, as a representative immune checkpoint, is often upregulated on the tumor cell membrane, promoting immune evasion.^[^ ^5d^ ^]^ The majority of research and drug development has focused on mPD‐L1, which always functions in a PD‐1‐dependent manner‐a classical tumor immune escape signaling pathway. However, anti‐PD‐L1 antibodies only block mPD‐L1, leaving non‐classical PD‐L1 forms unaddressed and resulting in suboptimal therapeutic efficacy.

The whole picture of PD‐L1 is more complex, with its expression in the cytoplasm, nucleus, and exosomes. We regard this property as “spatial heterogeneity expression”, which allows PD‐L1 to mediate other tumor invasion behaviors through PD‐1 independent pathways. This finding will reshape PD‐L1‐targeted therapy and herald the beginning of a new arena in the future. This heterogeneity of PD‐L1 presents a formidable challenge for the development of potent modulators, while the intricate interplay of regulatory mechanisms poses additional challenges for the development of upstream and downstream regulatory agents. We have analyzed the underlying regulation mechanisms of PD‐L1 in different subcellular locations, identifying the potential modulators such as degraders, downregulators and covalent inhibitors. These strategies may provide new approaches for targeting PD‐L1 therapeutically, although further investigation is needed for clinical translation. Building on the understanding of PD‐L1's spatial heterogeneity expression, researchers can further investigate how various tumor types regulate PD‐L1 and identify the most crucial factors in each tumor's development. Such insights will facilitate the development of personalized therapeutic strategies for cancer patients, as tumors may exhibit different PD‐L1 expression patterns. It also should be noticed that in this review although PD‐L1 is classified into mPD‐L1, cPD‐L1, nPD‐L1, and exoPD‐L1, the relationship between them is dynamic. For instance, after synthesis in the ER and Golgi apparatus, PD‐L1 can be transported to the cell membrane. mPD‐L1 can also enter cytoplasm through endocytosis to become cPD‐L1, or secrete into the extracellular space to become exoPD‐L1. Considering this feature of PD‐L1 expression, an area of further research is the development of combination therapies that simultaneously target mPD‐L1, cPD‐L1, and exoPD‐L1. Fortunately, researchers are cognizant of this issue and have developed multi‐targeted approach that acts simultaneously on various forms of PD‐L1, which possesses the potential to overcome the dilemma of current anti‐PD‐L1 antibodies and improve the overall therapeutic efficacy. For example, a prodrug, platinum covalently linked to BRD4 inhibitor JQ‐1, has been found to degrade PD‐L1 in both cytoplasm and membrane, thus reversing the immunosuppression microenvironment and suppressing tumor growth.^[^ ^134^ ^]^ A RNA interference nanoblocker, self‐assembly by peptide‐branched polymer and siRNA against PD‐L1, enables specific silencing of both mPD‐L1 and cPD‐L1 and obstructs tumor growth.^[^ ^135^ ^]^ Also, nanoparticle delivery strategies serve to change the subcellular localization of PD‐L1, thus enhancing the therapeutic efficacy. mPD‐L1 prones to recycle back to the cell membrane after internalized with antibodies binding. The PD‐L1 multivalent binding liposome design can deliver mPD‐L1 to lysosomes for degradation.^[^ ^136^ ^]^ In addition, we also note a new direction for future work. Compared to inhibitors, degraders seem to be a better option. PROTAC and LYTAC, as we mentioned in Section 3.2.1, have revolutionized the field of new drugs by hijacking the cellular ubiquitin‐proteasome or lysosome pathway for PD‐L1 degradation, bringing more insightful, broader, and longer‐lasting effects. They are completely different from small molecular inhibitors, which only bind to the active site on PD‐L1. Furthermore, PROTAC and LYTAC offer a transition from a occupied‐driven development paradigm toward an event‐driven and innovative development approach. Notably, For the development of degraders that both possess medical efficacy and biological safety, there are still some questions related to the side effects that remain unanswered. These conflicts including: (i) To what extent will the degradation of PD‐L1 trigger unexpected chain reactions within the body; (ii) Is the degradation of PD‐L1 an obstacle to effective long‐term anti‐tumor treatments; (iii) Does the degradation of PD‐L1 pose a challenge to any immunological memory. Thus, ensuring its safety and solving any potential issues demand further, intensive investigations.

In addition to targeting PD‐L1 itself, researchers can focus on developing agents targeting upstream and downstream regulatory pathways. Identifying key signaling molecules and understanding their role in PD‐L1 regulation will enable the development of drugs that can modulate PD‐L1 expression more precisely and effectively. The synthesis of novel compounds has the potential to provide versatile clinical benefits and will be the subject of future investigation.

In summary, the future of PD‐L1‐targeted cancer therapy will involve a multifaceted approach, incorporating a deeper understanding of the spatial heterogeneity expression of PD‐L1, biomarker identification, immune system modulation, combination therapies, resistance mechanisms, Al applications, and improved drug delivery. By exploring these avenues, researchers aim to develop more effective cancer immunotherapies and improve patient outcomes across various cancer types.

## Conflict of Interest

The authors declare no conflict of interest.
